# Immunological Microenvironment Diversity in Homogeneous and Non-Homogeneous Oral Leukoplakia

**DOI:** 10.3390/ijms27146111

**Published:** 2026-07-08

**Authors:** Ingrīda Čēma, Regīna Kleina, Madara Dzudzilo, Kristina Lasiené, Anita Dabužinskiene, Julianna Muceniece, Maksims Zolovs, Tālivaldis Freivalds

**Affiliations:** 1 Centre of Oral Medicine, Institute of Stomatology, Rīga Stradiņš University, Dzirciema Str. 20., LV-1007 Riga, Latvia; madara.dzudzilo@rsu.lv; 2 Department of Maxillo-Facial Surgery and Oral Medicine, Rīga Stradiņš University, Dzirciema Str. 16, LV-1007 Riga, Latvia; julianna.muceniece@rsu.lv; 3 Faculty of Medicine, Rīga Stradiņš University, Dzirciema Str. 16., LV-1007 Riga, Latvia; rkleina@inbox.lv; 4 Department of Histology and Embryology, Lithuanian University of Health Sciences, A. Mickeviciaus Str. 9, 44307 Kaunas, Lithuania; kristina.lasiene@lsmu.lt; 5 Institute of Anatomy, Lithuanian University of Health Sciences, A. Mickeviciaus Str. 9, 44307 Kaunas, Lithuania; anita.dabuzinskiene@lsmu.lt; 6 Statistics Unit, Rīga Stradiņš University, Dzirciema Str. 16., LV-1007 Riga, Latvia; maksims.zolovs@rsu.lv; 7 Institute of Life Sciences and Technology, Daugavpils University, Vienības Str. 13., LV-5401 Daugavpils, Latvia; 8 Faculty of Medicine and Life Sciences, Institute of Cardiology and Regenerative Medicine, University of Latvia, Jelgavas Str. 3, LV-1004 Riga, Latvia; freivald@latnet.lv

**Keywords:** oral leukoplakia, dysplasia, CD3, CD20, CD9, CD138 and CD68 proteins

## Abstract

Oral leukoplakia (OL) is a potentially malignant lesion, but only a proportion of cases undergo transformation to carcinoma (7.2% to 9.5%). The aim of this study was to analyze the possible differences and the role of immune cells in homogeneous and non-homogeneous OL at different grades of dysplasia. The infiltration density of T and B lymphocytes, macrophages, plasma cells, and cells expressing CD9 antigen was assessed semi-quantitatively in 50 OL cases using a 4-point scale—score 1 (<5%), score 2 (6–10%), score 3 (11–15%) and score 4 (16–20%)—via manual counting by two morphologists. Both clinical types of OL that showed dysplasia had noticeably higher proportions of CD138+ and CD68+ immune cells. Statistical analysis confirmed that only non-homogeneous OL with dysplasia showed an increase in the infiltration density of CD3+ and CD20+ lymphocytes. There was a statistically significant moderate positive correlation between the amount of CD9+ cells in the lamina propria and the number of labeled epithelial layers in OL of different thicknesses. Expression of CD138 and CD9 proteins in epithelial and connective tissue cells in OL indicates active epithelial–mesenchymal interaction during the premalignant stage of OL, but the expression of these markers in epithelial and immune cells was completely the opposite. The evaluation of CD3, CD20, CD9, CD138, and CD68 antigen expression, particularly in non-homogeneous leukoplakia, improves the accuracy of malignancy risk assessment in patients with dysplasia.

## 1. Introduction

Oral leukoplakia (OL) is the most prevalent oral potentially malignant disorder (OPMD), with an estimated global prevalence ranging from 3.41% to 11.74% [1,2,3]. Despite advances in clinical examinations and histopathological diagnostics, predicting which OPMDs will progress to invasive carcinoma remains a major clinical challenge. While OL is acknowledged as a precursor lesion, only a fraction of cases ultimately progress to malignancy [4,5]. It is currently known that a central role in this process is played by the microenvironment of oral leukoplakia, which includes local cellular, molecular, vascular, and immune surroundings that interact with the altered oral epithelium and influence whether the lesion remains benign, develops dysplasia, or progresses to carcinoma [6]. It should be noted that chronic inflammation can also act as an initiator and promoter of oral carcinogenesis, transforming normal oral mucosa into dysplastic tissue through persistent cellular stress and environmental alterations. Persistent inflammatory insults—whether from mechanical trauma, chronic infection, or habits such as smoking—trigger a cascade of molecular events that promote cellular proliferation, survival, and eventual malignant transformation [7]. In the assessment of oral epithelial changes and the risk of malignant progression in homogeneous and non-homogeneous leukoplakias, detection of the dysplasia stage remains the gold standard in histopathology [8]. The oral mucosa’s immune system maintains immunological balance with the help of salivary antimicrobial factors, epithelial barrier integrity, and resident immune cells [9,10] such as dendritic cells, natural killer cells, CD8+ cytotoxic T lymphocytes, mast cells, monocytes, macrophages, neutrophils, plasma cells, and B ly, which help the body fight against infections, detecting and eliminating dysplastic or virus-infected epithelial cells before malignant transformation occurs [11,12,13,14]. In the progression from healthy oral epithelium to dysplasia, and further to invasive oral carcinoma, the immune system plays an important role involving a complex interaction between epithelial cells and chronic inflammation [12,15]. The dysplastic cells actively remodel their surroundings from a state of immune surveillance to an immunosuppressive, hypoxic, and acidic “niche” that supports progression to cancer [15]. In recent years, increasing attention has been directed toward molecular biomarkers that may better reflect biological behavior and characterize the immunological microenvironment in OPMDs, particularly oral leukoplakia.

CD3 T cells are lymphocytes that are essential to the adaptive immune system, defined by the presence of the CD3 protein complex on their surface; they function primarily by facilitating T-cell receptor (TCR) activation, signal transduction, and TCR assembly, allowing T cells (CD4+ helper and CD8+ cytotoxic) to recognize antigens and initiate immune responses against pathogens or cancer [16,17,18]. Since the CD3 antigen is found on almost all mature T lymphocytes in humans, it serves as a reliable marker for identifying all T cells in immunohistochemical analysis of tissue samples [19] and in the context of oral epithelial dysplasia (OED) and oral squamous-cell carcinoma (OSCC) [20]. These T cells predominantly appear in the *stratum basale* and *stratum spinosum* of the epithelium, often in direct interaction with Langerhans cells (CD1a+) to recognize dysplastic keratinocytes [21]. Studies suggest that high densities of CD3-positive (CD3+) T cells in OPMDs, particularly in leukoplakia with dysplasia, indicate a protective immune response that may prevent malignant transformation. This means that the presence of CD3+ T cells in leukoplakia with dysplasia suggests an attempt by the immune system to recognize and eliminate abnormal cells, but reductions in (or absence of) these immune cells may facilitate the progression from dysplastic cells to cancer [6,21,22].

CD20 is a surface immune marker for B lymphocytes, expressed during all stages of B-cell development, from pre-B cells in the bone marrow through immature, naive, mature, and memory cells in lymphoid tissues and blood. Plasmablasts and plasma cells no longer express this marker [23,24]. Some T cells also can express CD20—mostly CD8+ effector memory T cells with pro-inflammatory features [25]. This marker is not typically expressed by normal oral epithelial cells themselves, but it may appear in low numbers in the subepithelial connective tissue as part of the normal immune surveillance [26]. B cells are also antigen-presenting cells (APCs); they secrete cytokines, provide regulatory molecules, and recognize antigens through the B-cell receptor complex on their cell membrane [24,27]. CD20 is used in research to evaluate the immune microenvironment of OED and its progression to oral squamous-cell carcinoma. Studies indicate that CD20+ B-cell infiltration increases in the subepithelial connective tissue stroma, often forming a cluster, as dysplastic lesions progress toward malignancy, reflecting a dynamic host immune response [16,28,29,30]. This establishes CD20 as a crucial indicator and points to its prognostic role in the microenvironment of dysplastic oral epithelium, with higher levels of infiltration correlating with higher grades of dysplasia [30].

CD9 protein belongs to the tetraspanin family and plays a role in various biological functions, such as cell adhesion, migration, membrane fusion, and signal transduction [31]. Available data suggests that CD9 expression may be preserved in oral leukoplakia regardless of the degree of epithelial dysplasia [32,33,34]; however, systematic comparisons between different clinical subtypes of leukoplakia, with or without dysplasia and invasive OSCC, remain limited [33,35]. In addition, there is limited understanding of how epithelial CD9 expression interacts with the surrounding immune environment in both leukoplakia and malignant lesions. Expression of CD9 antigen in cells of the lamina propria and squamous epithelium in both precancerous conditions and oral squamous-cell carcinoma is important in predicting their course, and even in treatment with targeted immunotherapy [35,36].

CD138, also known as syndecan-1 (SDC1), is a transmembrane proteoglycan that acts as a surface adhesion molecule, involved in cell adhesion and extracellular matrix modeling [37]; it is highly and specifically expressed on the surface of immature B cells and mature plasma cells, and it is used as a specific immunohistochemical surface marker to identify plasma cells and mature epithelial cells [38]. In tissues undergoing dysplastic changes, CD138 expression has been shown to be significantly reduced or weak in dysplastic oral epithelia compared to normal mucosa, with its intensity often decreasing as the severity of dysplasia increases [39]. This loss of adhesion molecule expression is a marker of early malignant transformation, making CD138 a potential indicator for assessing the risk of progression [40].

CD68 is a heavily glycosylated glycoprotein that is found abundantly in macrophages and other mononuclear phagocytes. Traditionally, the CD68 antibody is used as an immunohistochemical marker for pan-macrophage staining of inflamed tissues and for tumor tissue analysis [41]. Previous studies confirm the active role of macrophages in regulating immunosuppression, oncogenesis, and tumor progression in OPMDs, as well as during the progression to oral squamous-cell carcinoma [42,43,44]. A systematic review and meta-analysis conducted by Feltraco et al. [45] demonstrated a significant increase in the number of macrophages in the stromal subcompartment of OPMDs and indicated a correlation between macrophages and OPMD lesion severity. It has been found that increased densities of CD68+ macrophages are found in both the subepithelial and intraepithelial compartments of dysplastic lesions [44]; this indicates that CD68 acts as a potential biomarker for the progression of oral epithelial dysplasia.

The aim of the current study was to analyze the possible differences and role of immune cells in homogeneous and non-homogeneous OL at different grades of dysplasia.

## 2. Results

### 2.1. Descriptive Characteristics of the Analyzed Cases

The cohort included 50 oral leukoplakia patients. The mean age of the participants was 57.0 years (SD 14.1; range 18–75 years), with a non-significant male predominance (n = 29, 58.0% vs. n = 21, 42.0% female, *p* = 0.322). All oral leukoplakias were solitary, primary, and were located mostly on the buccal mucosa (n = 18, 36.0%) and the lateral side of the tongue (n = 17, 34.0%), followed by the floor of the mouth (n = 11, 22.0%). Other sites, including the lower lip and alveolar ridge, accounted for the remaining 8.0% (n = 4). Of all assessed cases of leukoplakia (OL), 18 (36.0%) were diagnosed as homogeneous OL, while 32 (64.0%) were identified as non-homogeneous types. Histopathological evaluation confirmed the presence of epithelial dysplasia in 33 cases (66.0%): 13 of them were mild, 7 moderate, and 13 severe; the new WHO classification [46] determined that 13 were low-grade and 20 were high-grade. The only type of dysplasia detected in homogeneous leukoplakia was low-grade, in seven cases (38.9% of homogeneous OL). Meanwhile, in non-homogeneous OL, dysplasia was low-grade in 6 cases and high-grade in 20 cases (21.9% and 62.5%, respectively). The prevalence of dysplasia was notably higher in non-homogeneous OL (26/32, 81.2%) compared to the homogeneous type (7/18, 38.9%).

#### Impact of Anatomical Localization on the Immune Microenvironment of Oral Leukoplakia

To determine whether the anatomic site of the lesion influences the immune microenvironment, we analyzed marker expression across the three primary sub-sites of oral leukoplakia: the buccal mucosa, lateral side of the tongue, and floor of the mouth. Kruskal–Wallis tests indicated no statistically significant differences in the median expression levels of CD20 (*p* = 0.240), CD138 (*p* = 0.154), or CD68 (*p* = 0.115) based on localization alone. Although CD3 expression showed a trend towards variation (*p* = 0.057), this did not reach the threshold for significance (Table 1).

### 2.2. Immunohistochemical Characteristics of Immune Cells in Oral Leukoplakia

**Figure 1:**
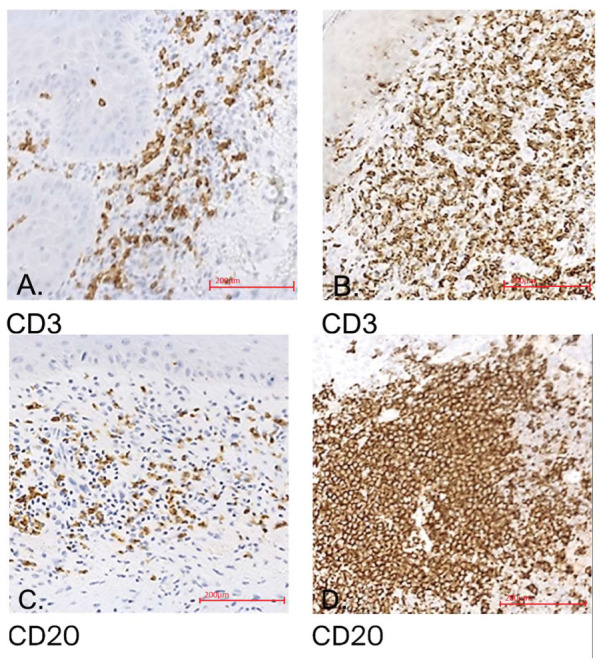
Representative immunohistochemistry of CD3 and CD20 antigens. (**A**,**B**) Increased CD3 expression from scores of 2 to 4 in OL with high-grade dysplasia. (**C**) B lymphocytes in the subepithelial area of homogeneous oral leukoplakia of score 2 with low-grade dysplasia. (**D**) Dense B-lymphocyte infiltration (score 4) in non-homogeneous oral leukoplakia. Immunoperoxidase, 200× magnification.

In the assessment of oral leukoplakia, we evaluated T and B lymphocytes, plasma cells, and macrophages/monocytes using the following markers: CD3, CD20, CD9, CD138 and CD68. The obtained kappa values (k = 0.87) indicated good agreement between the observers. In the control group with healthy mucosa, only a small number of T lymphocytes were detected beneath the basement membrane and in the epithelial layer up to the keratinized regions on the surface of the OL. In OL, CD3 lymphocytes were found to be predominantly situated in the subepithelial zone, with a diffuse infiltration pattern observed. The density of CD3-labeled lymphocytes in homogeneous oral leukoplakia, both with and without dysplasia, showed no statistically significant difference (*p* = 0.894). In non-homogeneous oral leukoplakia without dysplasia, T-lymphocyte infiltration typically had a score of 2 (Figure 1A); however, when dysplasia was present, the infiltration density increased, with 50% of cases reaching a score of 4 (*p* < 0.001) (Figure 1B).

In our analyzed cases of healthy mucosa, B lymphocytes were absent in the epithelial layer and exceedingly rare in the *lamina propria*. Infiltration with B (CD20) lymphocytes was predominant in homogeneous OL at score 1. When dysplasia was present in this type of OL, there were no observed changes in the density of CD20-positive cells. Although in 3 out of 7 cases the B-lymphocyte infiltration density increased until score 2 (Figure 1C), statistical analysis of the results did not show a statistically significant difference (*p* = 0.284). In non-homogeneous OL without dysplasia, the density of CD20+ cells reached score 1, similar to homogeneous OL. The immune response involving B lymphocytes was primarily evident as small infiltrates around capillaries. In cases of non-homogeneous OL with dysplasia, B-lymphocyte infiltration was variable and reached a score of 4 in 38% of cases (Figure 1D). These differences were statistically significant (*p* < 0.01).

**Figure 2:**
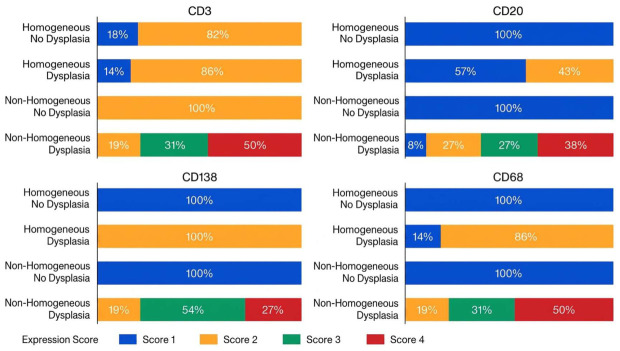
Distribution of semi-quantitative immunohistochemical staining scores. Stacked bars show the relative proportion of patients exhibiting expression scores from 1 (lowest) to 4 (highest) across the clinical types of OL, with and without dysplasia (Table S2).

In the immune microenvironment of non-homogeneous leukoplakia with dysplasia, the T- and B-cell infiltration densities were synchronous with changes in plasma cell and macrophage responses (Figure 2).

**Figure 3:**
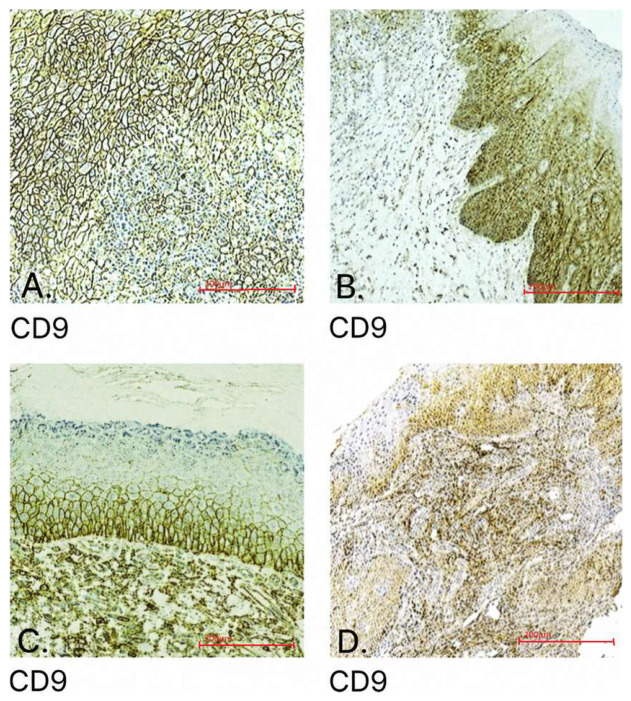
Representative immunohistochemistry of CD9 antigen. (**A**) CD9 is overexpressed on the membranes of hyperplastic squamous epithelium and certain mononuclear cells in homogeneous OL. (**B**) Expression within the membranes and cytoplasm of dysplastic squamous epithelium in non-homogeneous oral leukoplakia. (**C**) Abundant CD9+ immunocompetent cell infiltrate beneath homogeneous OL. (**D**) Synchronous increased expression of CD9 antigen on dysplastic epithelium and in the fibrosis zone of the lamina propria beneath non-homogeneous OL. Immunoperoxidase, 200× magnification.

**Figure 4:**
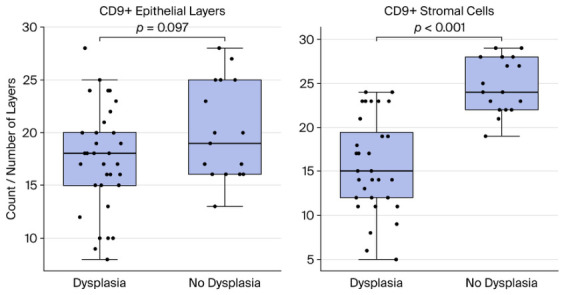
Comparison of CD9 antigen expression in dysplastic vs. non-dysplastic lesions of oral leukoplakia (Table S1).

In morphologically unchanged mucosa, CD9 protein expression was only in epithelial membranes, on average in 5 ± 1.1 layer. In oral leukoplakia, CD9 antigen was absent in the superficial layers and in regions containing keratohyalin granules. Cell membranes were slightly corrugated when labeled with CD9 antigen (Figure 3A), which remains present on the membrane of epithelial cells in areas with low-grade dysplasia. In instances of high-grade dysplasia, CD9 protein was additionally found inside the cytoplasm (Figure 3B). The number of CD9-marked epithelial layers in OL was somewhat lower in cases featuring dysplastic lesions (17.5 ± 5.0 vs. 19.9 ± 4.6), although this difference did not reach statistical significance (*p* = 0.097) (Figure 4).

Within the lamina propria, the CD9 antigen was detected not only on macrophages but also on monocytes, some B and T lymphocytes, lymphatic endothelial cells, and within fibrotic areas of the lamina propria (Figure 3C,D). In healthy mucosa, there are an average 5 ± 1.01 mononuclear cells in one field of vision at 400× magnification under the basal membrane. In oral leukoplakia without dysplasia, there were 24.8 ± 3.1 CD9-labeled mononuclear cells in the subepithelial zone, but with dysplasia there were 15.8 ± 5.4 (*p* < 0.001), and these were mainly non-homogeneous leukoplakia cases.

**Figure 5:**
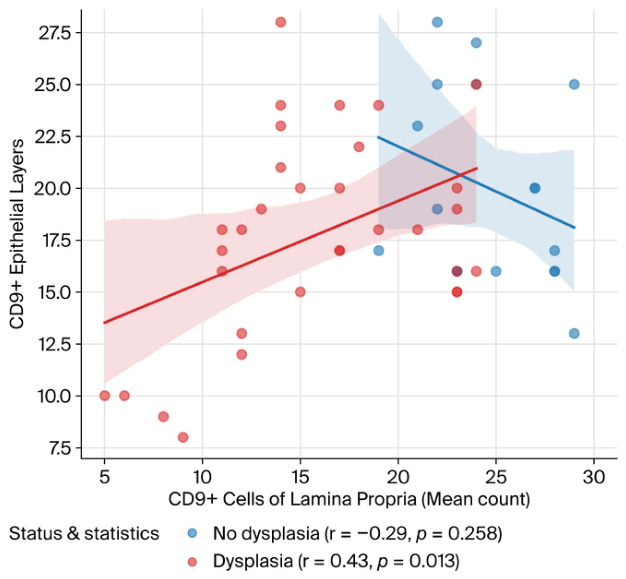
Scatterplots with linear regression lines and shaded 95% confidence intervals illustrating the link between epithelial and lamina propria cells labeled with CD9 antigen (Table S1).

Evaluation of the expression of CD9 protein uncovered a fundamental shift in the epithelial and immune microenvironment interactions in the lamina propria under OL. Among cases of oral leukoplakia with dysplastic changes, there was a moderate yet statistically significant positive relationship between the number of CD9-labeled epithelial layers and the number of CD9-positive immune cells (r = 0.43, *p* = 0.013). Conversely, in oral leukoplakia without dysplasia, no significant correlation was found between these values (r = −0.29, *p* = 0.258) (Figure 5).

**Figure 6:**
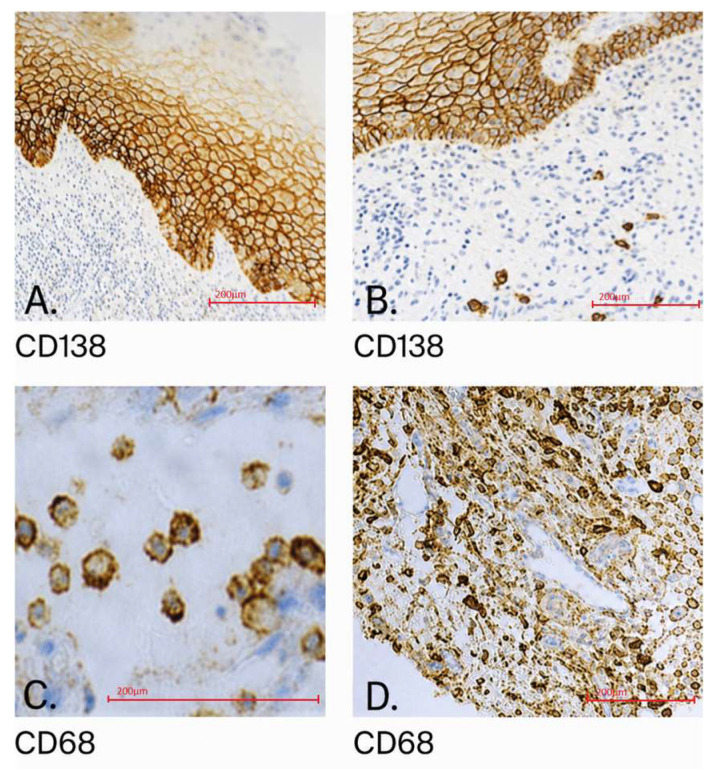
Representative immunohistochemistry of CD138 and CD68 antigens. (**A**) CD138 antigen deficiency in superficial squamous epithelial areas with keratinization and keratohyalin granules; 200× magnification. (**B**) Antigen expression in both epithelial and plasma cells (score 1) in a homogeneous OL without dysplasia; 200× magnification. (**C**) Macrophage density score of 2 beneath homogeneous OL; 400× magnification. (**D**) Dense macrophage infiltration (score 4) within the deeper zone of the lamina propria in non-homogeneous leukoplakia; immunoperoxidase, 200× magnification.

CD138 antigen was found on plasma cells, the endothelium of lymphatic capillaries, and the membranes of squamous epithelial cells in both normal tissues and oral leukoplakia. However, regions with keratinization and keratohyalin granules lacked CD138 expression in the epithelial cells (Figure 6A). In normal mucosa, the density of plasma cells in the lamina propria was at the lower limit (score 1). In cases of homogeneous leukoplakia, the number of CD138+ cells showed only a minor increase (from score 1 to score 2) when dysplasia was observed (Figure 6B). In contrast, in non-homogeneous OL, the plasma cell response increased rapidly with the appearance of dysplasia, and 81% of patients showed infiltration of score 3 or 4 (*p* < 0.001). In both clinical types of leukoplakia without dysplasia, there was no statistical difference in the density of plasma cell infiltrates (score 1). Meanwhile, the difference in the density of plasma cell infiltrates between homogeneous and non-homogeneous leukoplakia with dysplasia was statistically significant (*p* < 0.001).

The CD138 marker demonstrated that the plasma cell infiltrate was diffuse and often located beneath the lymphocyte layer rather than directly beneath the basement membrane. In homogeneous oral leukoplakias, low-grade dysplasia did not affect syndecan-1 expression in squamous epithelium, as the number of epithelial layers labeled with CD138 antigen did not decrease. In non-homogeneous leukoplakias, both low-grade and high-grade dysplasia showed reduced CD138 antigen expression, with 11% of cases testing negative. In non-homogeneous OL, we often diagnosed asynchronous expression of CD138 antigen in epithelial and immune cells, with decreased expression in dysplastic epithelium, while the density of labeled plasma cells rose to scores of 3 and 4.

Subsequently, we evaluated the quantity, distribution, and infiltration pattern of macrophages using the CD68 antigen. Furthermore, employing the CD9 marker enabled us to gather additional insights into the interactions between mononuclear cells in the lamina propria under leukoplakia, both with and without dysplasia. Healthy mucosa contains only a small number of macrophages per field at 400× below the basement membrane (Figure 6G). In homogeneous oral leukoplakia (OL) without dysplasia, every case showed a macrophage density score of 1. However, when dysplasia was present, 86% of OL cases exhibited a macrophage density score of 2 (*p* = 0.05). In contrast, in non-homogeneous OL without dysplasia, all cases showed a mild macrophage reaction (score 1), but when dysplasia was diagnosed, the scores were different, with score 2 in 19%, score 3 in 31%, and score 4 in 50% of cases (*p* < 001) (Figure 6D).

### 2.3. General Linear Model and Interaction Analysis

The GLM constructed for CD3, CD20, CD138, and CD68 demonstrated large effect sizes (partial η^2^ = 0.588–0.794), indicating strong associations between predictors and immune marker expression (Table 2).

The primary analysis revealed a consistent and statistically significant interaction between the presence of dysplasia and clinical types of OL across all four markers (*p* < 0.05). This indicates that the immune response, which involves lymphocytes, plasma cells, and macrophages, is connected to the degree of dysplasia in both types of lesions (homogeneous vs. non-homogeneous). As a result, simple effects analyses were carried out to break down these interactions.

### 2.4. Characterization of the Interaction Among Clinical Forms of Leukoplakia, Dysplasia, and Immune Cell Infiltration Density Scores

Analysis of the interaction effects showed that the increase in immune markers within dysplastic tissue is mainly—and for certain cell types exclusively—due to non-homogeneous leukoplakia (Table 3).

A marked rise in CD3 and CD20 marker expression linked to dysplasia was observed exclusively in non-homogeneous leukoplakia. In non-homogeneous lesions, dysplasia resulted in a mean increase of 1.31 units for CD3 (95% CI 0.92–1.67, *p* < 0.001) and 1.95 units for CD20 (95% CI 1.49–2.38, *p* < 0.001). Conversely, within homogeneous OL, neither marker showed a statistically significant deviation from non-dysplastic baseline levels (CD3: estimate 0.043, 95% CI −0.36–0.42, *p* = 0.894; CD20: estimate 0.419, 95% CI 0.006–0.95, *p* = 0.284). Dysplasia showed much higher CD138 and CD68 expression in both clinical types of leukoplakia, with non-homogeneous OL displaying approximately twice the increase. For CD138 protein, the average difference associated with dysplasia in non-homogeneous lesions was roughly twice as large as that found in the homogeneous subgroup (estimate 2.08 [95% CI 1.76–2.39] vs. 1.00 [95% CI 0.80–1.18], respectively; *p* < 0.001 for both). Similarly, CD68 expression demonstrated a more robust response in non-homogeneous leukoplakia (estimate 2.34, 95% CI 1.95–2.66, *p* < 0.001) compared to the moderate increase seen in homogeneous OL (estimate 0.89, 95% CI 0.51–1.18, *p* = 0.005). After accounting for demographic variables such as age and gender, no significant differences were found in the expression levels of any of the four markers (*p* > 0.05 across all types of leukoplakia).

Beyond the comparison of mean values, an analysis of the semi-quantitative staining distribution revealed a fundamental shift in the immune profile depending on the clinical form of OL (Figure 3). In homogeneous leukoplakia, the immune response was generally low even with dysplasia; most cases showed scores of 1 or 2, with no instances of score 4. In sharp contrast, the non-homogeneous leukoplakia demonstrated a distinct polarization associated with dysplasia. Non-dysplastic lesions displayed a varied pattern, whereas dysplasia arising in non-homogeneous lesions was marked by a notable increase in infiltrate density. Specifically, for CD138 and CD68 antigens (plasma cells and macrophages), the dysplastic non-homogeneous cohort exhibited a marked dominance of score 4, suggesting that this clinical type of leukoplakia represents a distinct, high-grade immune microenvironment state rather than a mere linear progression of the baseline immunity.

## 3. Discussion

One of the most common clinically diagnosed OPMDs is leukoplakia, which in each specific situation requires careful clinical and morphological evaluation to prevent progression to malignant transformation in a timely manner. Scientific articles demonstrate that homogeneous leukoplakia is the most common clinical form of OL, often representing over 80–95% of cases in clinical studies, reflecting no dysplastic changes [47,48,49,50]; The data show that homogeneous leukoplakia has a lower and slower rate of transformation, with an overall lifetime malignant transformation rate of 8.6% [3], but it is not completely risk-free, so constant monitoring is necessary. According to Tenore et al. [49], the homogeneous type is most common, followed by the verrucous and speckled types of non-homogeneous leukoplakia, which is consistent with our observations, where the speckled subtype (*erythroleukoplakia*) was most common. Power analysis indicated that, despite the relatively small sample size, the interaction between dysplasia and the clinical form of OL demonstrated substantial effect sizes, with partial η^2^ ranging from 0.17 to 0.23. Post hoc power estimates indicated moderate-to-high statistical sensitivity (0.80–0.90), suggesting that the study was sufficiently sensitive to detect interaction effects of the magnitude observed. The fact that non-homogeneous leukoplakia carries a higher risk of malignant transformation to invasive carcinoma due to the increase in the severity of dysplastic features has been noted by several authors [48,50,51]. The meta-analysis performed by Aguirre-Urizar et al. [52] demonstrated a 4.06-fold increased risk of malignization for non-homogeneous OL, but a 23.8-fold increased risk in the presence of oral epithelial dysplasia.

The location of oral leukoplakia did not influence the immune cell density in homogeneous or non-homogeneous OL, likely due to the study’s small sample size. Crucially, the interaction pattern between dysplasia and immune upregulation was consistent across all anatomic sites. Dysplasia consistently correlated with higher immune marker scores, no matter whether the lesion appeared on the tongue, the buccal mucosa, or the floor of the mouth. This indicates that the immune response is mainly prompted by dysplastic changes rather than differences in local tissue environments. The gender of the patients did not affect the frequency of dysplasia, nor did the characteristics of the immune environment in homogeneous and non-homogeneous OL (*p* = 0.322).

Our study demonstrates highly variable cellular immunity around two clinically distinct forms of OL. Taken together, the combined evaluation of CD3, CD20, CD9, CD138, and CD68 underscores the interplay between epithelial dysregulation and immune microenvironment remodeling in premalignant oral lesions.

T and B lymphocytes are important participants in the immune processes of oral mucosa. The reaction of pan-T lymphocytes (antigen CD3) in the analyzed OL cases was more pronounced than the B-cell response. Under homogeneous leukoplakia, in the lamina propria, the T-lymphocyte density reached a score of 2 and was not statistically significantly affected by the presence of mild dysplasia, similar to the reaction of B cells. In contrast, in non-homogeneous leukoplakia with dysplasia, the T-lymphocyte density increased significantly in 81% of patients, and the difference was statistically significant (*p* < 0.001). Both original research and literature reviews on oral leukoplakia and OSC carcinoma have demonstrated variable functions for different subtypes of T cells. Öhman et al. [21] used confocal laser scanning microscopy to reveal co-localization of Langerhans cells (LCs) and T cells in OL with dysplasia and in OSCC, concluding that this reflects an ongoing immune response against cells with dysplasia and malignant transformation in oral leukoplakia. However, in oral cancer research, Hladikova et al. [53] revealed that CD8+ T-cell and B-lymphocyte interactions can predict patients with a good prognosis.

In our study, B lymphocytes were absent in healthy mucosal tissue. Data regarding CD20-expressing lymphocytes in OL are inconsistent. Our study showed that, in homogeneous and non-homogeneous OL without dysplasia, the density of infiltration with B lymphocytes did not differ, but it was present at score 1, was along with the immune cell reaction to long-term hyperplasia and keratinization processes within them. However, with the onset of dysplasia, the intensity of their infiltration increased, which is consistent with the results of Gannot et al. [28], who found that B lymphocytes increase most rapidly as the tongue epithelium moves from hyperkeratosis through various degrees of dysplasia to squamous cell carcinoma. In cases of low-grade dysplasia, immature epithelium occupies one-third of the thickness of the layer, and B and T lymphocytes do not respond to this slightly altered number of epithelial cells. With the development of high-grade dysplasia in non-homogeneous OL, B-lymphocyte infiltration becomes variable. The density of infiltration ranged from score 1 to score 4, but 65% of cases had a score of 3 or 4. This may indicate the different clinical courses of non-homogeneous OL: dysplasia may progress to cancer in situ or remain at the previous level. Our reasoning is that B lymphocytes play two roles—a topic that has been thoroughly examined in the scientific literature: a comparative analysis of publications allows the authors to conclude that B lymphocytes may either suppress or promote tumor growth [28,54,55]. Debates over their anti-tumor and pro-tumor effects also continue in studies on oral cancer [56,57,58]. Further research is required in order to better understand how B-cell subtypes affect the progression of oral leukoplakia to carcinoma.

CD9 antigen was expressed in the oral mucosal epithelium and in the cells of the lamina propria beneath OL. It was analyzed on serial histological sections, counting the numbers of epithelial layers and connective tissue cells expressing this marker in three areas beneath the basement membrane of OL. In cells such as epithelium, endothelium [59], macrophages [31], lymphocytes [60], and fibroblasts [61], tetraspanin is marked by a single CD9 antibody, which is also used together with CD81 and CD63 to demonstrate proteins that indicate sites of exosome accumulation in tissues. There has not been much immunohistochemical research on CD9 antigen changes in OPMDs and its role in oral precancerous lesions with epithelial dysplasia [32,33,59,62]. Scientists have focused on studying the role of CD9 protein in the epithelium [33,36], but only a few groups of authors have evaluated its presence and significance in the lamina propria of the oral mucosa beneath OL [31,36,63]. Under a light microscope, CD9 antigen expression appears as irregular, thickened lines on the membrane [64,65]; additionally, CD9 has been recognized as a mediator of intercellular communication [66,67], which we propose also occurs among malignant cells—except in cases that show complete CD9 loss in carcinomas [32]. In our analyzed cases, CD9 antigen was strongly expressed in the epithelial membrane of OL without dysplasia, but its translocation into the cytoplasm of dysplastic epithelium points to the involvement of organelles in this process. The number of epithelial layers labeled with the CD9 antigen was not significantly different between homogeneous and non-homogeneous leukoplakia, because the epithelial layer in homogeneous OL is relatively intact, dominated by hyperplasia and hyperkeratosis. In low-grade dysplasia, as usually only one-third of the oral mucosa shows architectural changes and immature epithelium, it was diagnosed in only 33.3% of cases. However, in non-homogeneous OL with high-grade dysplasia (62.5% of cases), we observed a noticeable translocation of the CD9 antigen from membranes to cytoplasmic structures. In this study, the overall increase in the total amount of membrane-bound and cytoplasmic CD9 protein in non-homogeneous OL with high-grade dysplasia indicates its involvement in progression toward carcinoma. This is consistent with the findings of Baghban et al. [67], who proposed that CD9 protein may promote tumor growth, as cancer cells can secrete vesicles carrying molecular cargo that modifies the tumor microenvironment to support metastasis. Buim et al. [63] found that disease-free survival and 5-year overall survival in carcinoma patients with downregulated or negative CD9 expression were significantly lower than in patients with positive CD9 protein expression. Other scientists have also mentioned the disappearance of the CD9 protein in malignant keratinocytes of the oral mucosa and linked it to tumor dedifferentiation and its metastatic process [32]. A comparison of serial histological specimens from our study with the investigated markers demonstrated that CD9 protein was found on B lymphocytes, macrophages/monocytes, plasma cells, and more rarely on some T-lymphocyte subsets and fibroblasts, but this does include all of the cells involved in the immune microenvironment under leukoplakia. The number of CD9+ cells in one field of vision (400×) in the subepithelial zone of homogeneous leukoplakia is almost five times larger than in healthy mucosa. The latter is characterized by an intense immune-competent cell response to epithelial hyperplasia and mild, reversible dysplasia. CD9 antigens decrease in number as the dysplasia severity increases in non-homogeneous OL. Conversely, Shetty et al. [68], in a detailed review on head and neck squamous-cell carcinomas, concluded that the CD9 antigen can be both a tumor suppressor (when lost on epithelial cells) and a tumor promoter (when present on lamina propria cells), as these proteins form multimeric complexes with one another and other cell-surface proteins—including integrins, leukocyte antigens, and signaling molecules—at specialized tetraspanin-enriched microdomains. The authors found that CD9’s role as a marker of tumor inhibition or tumor progression depends on the molecule that interacts with this antigen. Infiltration of the subepithelial zone with mononuclear CD9-labeled cells was intense in homogeneous OL, which we concluded was due to a rich immune cell response to hyperplasia, keratinization, and mild dysplasia processes within them. This rich immune response possibly slows down the progression of mild dysplasia to a more severe form or even ensures its regression. According to data from the literature, even moderate dysplasia in oral leukoplakia may regress in 85–97% of cases [69]. In both clinical types of OL, CD9 protein was also expressed in the endothelium of lymphatic vessels. The synchronous expression of tetraspanin in epithelial cells, lymphatic endothelial cells, and lamina propria cells indicates close interaction among the squamous epithelium, immune cells, and the microcirculatory network in the subepithelial zone of the oral mucosa [61,67]. CD9 antigen was identified in fibroblasts of the lamina propria in 17% of non-homogeneous OL cases with high-grade dysplasia. Most of these cases were identified when the density score of immune cells dropped from 4 to 2, resulting in fibrosis of the lamina propria, which intensifies tissue hypoxia, worsens trophism in the connective tissue under the epithelium, and thereby promotes epithelial cell dedifferentiation in leukoplakia with high-grade dysplasia. Dynamic changes in the immune microenvironment of oral leukoplakia can only be accurately demonstrated by repeated biopsy examinations, which are not always possible.

Another antigen whose expression we analyzed in OL epithelium and plasma cells was CD138 (syndecan-1), a heparan sulfate proteoglycan with three domains: extracellular, transmembrane, and cytoplasmic. Importantly, biochemists have proven that the molecular weight of syndecan-1 varies significantly due to its glycosylation from 30–80 kDa up to 200 kDa, whether it is the core protein or the full proteoglycan [70]. We consider that this may affect the intensity of antigen expression. Different biochemical forms of CD138 are crucial for squamous epithelium development, adhesion, and migration in benign and malignant processes. The structure allows syndecan-1 to act as a receptor, binding to various growth factors, extracellular matrix components, and cytokines via its heparan sulfate chains. In the epithelium of oral leukoplakia, most scientists agree that CD138 antigen expression decreases. Soukka et al. [71], Lakkam et al. [72], Tegginamani et al. [40], and Akkaloori et al. [39] showed that CD138 is gradually lost as the severity of dysplasia progresses. The findings of our study are fully consistent with the data of the authors mentioned above. Our opinion is that the loss of the CD138 antigen is associated with impaired maturation and differentiation of the oral squamous epithelium, leading to the formation of dysplasia. In oral squamous-cell carcinoma, an analogous trend is observed, as syndecan-1 expression levels correlate with the differentiation grade of cancer. Basharat et al. [73] proved that the loss of CD-138 expression in oral squamous -cell carcinoma is also associated with tumor aggressiveness and poor prognosis. Most analysis of CD138+ plasma cells by researchers have been carried out in oral inflammatory processes [74] and malignancies such as multiple myeloma [74] and OSCC. Máthé et al. [74] concluded that CD138 expression in lamina propria cells proved to be a significant risk factor for recurrence and tumor-specific death within a 24-month period after surgery. Meanwhile, Mukunyadzi et al. [75] concluded that increased syndecan-1 expression in connective tissue cells may contribute to tumor cell invasion and the development of metastases in head and neck carcinomas. For an accurate prediction of the clinical course of OL, the detection of CD138 antigen in the epithelium must be accompanied by an analysis of its expression in plasma cells, but there are very few such reports. As far back as 1979, Loning and Burkhardt [76] used an immunoenzymatic method to find that the incidence of immunoglobulin (IgA and IgG)-labeled plasma cells was twice as high in cases of leukoplakia where dysplasia was present. In the oral mucosa, these molecular reactions play an important role both in squamous epithelium and in the subepithelial zone, by interacting with other immune-competent cells. In our analyzed group of cases, CD138 antigen under the basement membrane was expressed on the plasma cells and endothelium of lymphatic vessels in healthy oral mucosa and in both clinical subtypes of OL, with and without dysplasia. While no dysplasia was detected in either clinical type of leukoplakia, the infiltration density of plasma cells did not differ (score 1). If we analyze the homogeneous OL cases separately, we can see that the dysplastic process affects the infiltrate density in a very stereotypical way: in most patients, it changed from score 1 to score 2 when low-grade dysplasia was diagnosed. The fact that non-homogeneous OL without dysplasia also only shows mild infiltration with plasma cells (score 1) is clinically important. In non-homogeneous leukoplakia with dysplasia, the density of plasma cells in the subepithelial zone was variable, whereas intensive infiltration prevailed: in 81% of cases, the score was 3 and 4, but the grade of dysplasia in this type of OL also varied low-grade in 21.9%; high-grade in 62.5%). Simple effects analysis of dysplasia on immune marker expression, stratified by clinical form, showed that infiltration by plasma cells correlates with dysplasia grade specifically within the non-homogeneous subgroup, as the estimated effect of dysplasia was 2.075, as opposed to 0.999 in the homogeneous subgroup. The expression of CD138 in the oral mucosa should be assessed in a complex manner, in both epithelium and plasma cells together. Thus, based on the results obtained and data from the literature, we can conclude that the loss of CD138 antigen expression in OL epithelium with high-density infiltration of plasma cells in the *lamina propria* indicates an increased risk of developing malignancy. Since plasma cells are the most important producers of antibodies such as immunoglobulins, it would be valuable to evaluate IgA and IgG in oral leukoplakia.

In recent years, modern studies of the oral mucosa have been carried out on its immunoglobulins A, G and M in saliva and gingival crevicular fluid [77,78], which undoubtedly reflect the functional activity of plasma cells not only in small mucosal lesions of a few square centimeters, such as oral leukoplakia, but also in the entire 200 sq. cm oral mucosa and the salivary glands. Increased levels of salivary immunoglobulins are often observed in oral potentially malignant disorders (OPMDs) and oral squamous-cell carcinoma (OSCC), indicating a local immune response to epithelial changes.

Next, we analyzed macrophage infiltration density and location, from healthy mucosa to various clinical types of leukoplakia, with and without dysplasia. The transmembrane glycoprotein CD68 acts as a marker for all types of macrophages, but there are studies showing that this antigen is more frequently expressed on M1 macrophages [79], while CD163 and CD20 antibodies mark M2 macrophages [80]. The oral mucosa contains M1 macrophages that display pro-inflammatory properties and play an anti-tumor role; conversely, M2 macrophages demonstrate anti-inflammatory features and have a pro-tumor profile [67]. In recent years, in vitro studies have shown that M2 cells can be further divided into M2a, M2b, M2c, and M2d subtypes [80]; as antigen-presenting cells, they mediate immune responses to the oral microbiota, food, and other antigens, but they can also function within microenvironments of any pathological lesion of the oral mucosa. In oral pathology, Wang et al. [79] found that M1 macrophages can polarize into the M2 variant. In the healthy mucosa of the control group, there were few CD68-positive cells under the basement membrane. In intact oral mucosa, macrophages are essential for innate immunity, but with the development of leukoplakia, adaptive local immunity processes start. The density of macrophage infiltration in OL with low-grade dysplasia showed scores of 1 and 2; the difference was statistically significant (*p* = 0.005), and the effect of dysplasia was 0.885. The influence of interactions between epithelial and immune cells will continue if the leukoplakia is not removed in time. The differences in OL macrophage infiltration densities were more pronounced in non-homogeneous OL. Accordingly, in OL with and without dysplasia, CD68-labeled cells ranged from scores of 1 to 4, which points to a heterogeneous picture of immune responses in non-homogeneous OL, with a potentially variable clinical course. The large density of macrophages may influence the progression of high-grade dysplasia to malignancy, as also proven by Chistiakov et al. [41] and Sutera et al. [44]. The study of Hanania et al. [81] suggests that macrophages, especially the M2 type, stimulate the transformation of high-grade dysplasia into cancer [81]. Researchers have proven that activated CD163+ macrophages may secrete matrix metalloproteinase–9, which degrades the basement membrane and it fundamental for invasive tumors. Studies by Weber et al. [43] and Feltraco et al. [45] found a significant rise in M2 macrophages in OL cases with malignant transformation within five years compared to those without. However, the research of Sutera et al. [44] proved that, in OL with severe dysplasia, macrophages begin to produce cytokines that promote epithelial instability [44]. The antigen expression on macrophages is a highly regulated process, as shown by the study of Shigeoka et al. [82], in which M2 macrophages were labeled with CD163 and CD206 antigens, but only the CD163 protein showed a correlation between clinical morphological data and the grade of dysplasia in leukoplakia. By analogy, in oral squamous-cell carcinomas, strong macrophage infiltration has been shown to correlate with advanced malignancy, lower differentiation grade, and metastasis. Data from the literature show that OSCC samples showed the highest macrophage infiltration and strongest M2 polarization [71]. Tumor-associated macrophages act in a tumor-promoting manner through immunosuppression, angiogenesis, and the promotion of cancer cell invasion [83].

It should be highlighted that, in non-homogeneous leukoplakia, infiltration with lymphocytes (B and T) increases alongside plasma cells and CD68 macrophages, indicating a coordinated immune microenvironment response under this type of leukoplakia. To a certain extent, the density of infiltration is also influenced by the duration of leukoplakia—as noted by patients in their medical history—and the condition of the microbiota in the mouth [84,85].

### Limitations of the Study

Regarding the limitations of the present study, it should be noted that the amount of oral leukoplakia was relatively small because we analyzed only the data from patients consulted in our center. Although the number of immune cells expressing CD3, CD20, CD138 and CD68 proteins in non-homogeneous oral leukoplakia is statistically significant compared to homogeneous OL, the data obtained and evaluated with caution, due to the small number of cases. It is possible that the expression of CD9 and CD138 markers in the epithelium and lamina propria cells of leukoplakia is also unique to this group of non-homogeneous OL, which includes various clinical variants of it. Our study used only the traditional lymphocyte markers CD3 and CD20. In the future, supplementary studies of T- and B-lymphocyte subtypes should be carried out to clarify the correlation of their infiltration density with the severity of dysplasia in homogeneous and non-homogeneous OL. CD9 antigen studies should be combined with electron microscope studies of exosomes, which would open up more possibilities for the interaction of immune cells with squamous dysplastic oral epithelium. In retrospective studies, it was not always possible to evaluate etiological factors and these limitations may only be improved by the prospective enrollment of patients with different types of OL. Future multicenter longitudinal studies will be needed to confirm our findings on the role of the CD3, CD20, CD9, CD138, and CD68 antigen combination in the immune environment of oral leukoplakia.

## 4. Materials and Methods

### 4.1. Study Group

Fifty patients with homogeneous and non-homogeneous oral leukoplakia were consulted and biopsies were taken at the Oral Medicine Centre of the RSU Institute of Stomatology between 2017 and 2023. All patients had previously given their consent for the data to be used for the study (Decision No 3/18 August 2016 of the Ethics Committee of RSU). Radical surgical excision of oral leukoplakia within visually unaffected margins was performed in the Oral and Maxillofacial Surgery Centre of P. Stradiņš Clinical University Hospital (PSKUS) and the material was sent to the Pathology Department, where the material blocks were archived. The comparative group consisted of 20 patients with benign lesions, whose intact distal and proximal regions were used as normal mucosa samples. The presence of intact non- keratinized mucosa was confirmed microscopically.

Samples from the obtained paraffin blocks were cut into 4-micron-thick sections, which were stained with hematoxylin and eosin and then assessed under a light microscope. The clinical type (homogeneous/non-homogeneous), degree of dysplasia, and amount, location, and pattern of *lamina propria* cell infiltrates under leukoplakia were evaluated. To evaluate homogeneous leukoplakia, the following clinical parameters were considered: uniformly white, well-demarcated, flat, and thin appearance (some may be thick); smooth, wrinkled, or fissured (corrugated) surface texture; may show shallow cracks; asymptomatic [86]. There were n = 18 samples corresponding to this category of leukoplakia (*leukoplakia simplex*). To evaluate clinically non-homogeneous leukoplakia, we considered the following parameters: appearance of non-uniform white or mixed red-and-white patches; irregular surface; granular, nodular or verrucous texture; irregular or indistinct borders [87].

There were n = 32 samples corresponding to this category (*erythroleukoplakia* n = 17; *verrucous* n = 11; *nodular* n = 4). Since the individual subtypes of non-homogeneous leukoplakia were small in number, we used only the total number of non-homogeneous leukoplakia in our study.

We evaluated the morphology of oral leukoplakia samples by identifying tissues with and without signs of dysplasia. For this purpose, we used the binary system recommended by the WHO, dividing the dysplastic leukoplakia into low-grade (mild/moderate) and high-grade (severe/CIS) [46]. Immune cell expression was evaluated under each type of dysplasia.

### 4.2. Immunohistochemical Examination

Immunohistochemical visualization of the antigens of interest was performed on the same formalin-fixed, paraffin-embedded consecutive sections of oral leukoplakia and healthy tissues. We evaluated the infiltration of immune cells expressing the CD3, CD20, CD138, and CD68 antigens as 4 scores of labeled cells, using a semi-quantitative method adapted from the study of Nankivell [33]. Two individual raters (R.K. and K.L.), with extensive experience in immunohistochemistry assessment, independently scored each sample. These scientists were blinded to the clinical diagnosis of OL. The interobserver agreement between the two independent morphologists was assessed using the Cohen’s kappa coefficient for the 4-point ordinal scale [88]. They manually performed the cell counts, recording the percentages across three fields of view at 400× magnification: score 1 (<5%), score 2 (6–10%), score 3 (11–15%), and score 4 (16–20%); the arithmetic mean was then calculated. Immune cells were counted under the basal membrane of the leukoplakia on both its lateral sides and in the center. To more accurately evaluate the CD9 antigen, we determined its expression in three fields of view at 400× magnification in epithelium and in immune cells of the *lamina propria*.

Antigen expression in immunohistochemistry was assessed using a standard polymer-based visualization system—specifically, the EnVision method from Dako, Glostrup, Denmark/Agilent, Ejby, Denmark. For immunohistochemistry, slides were incubated with 3% H_2_ O_2_ for 10 min to block endogenous peroxidase and protein activity. The microwave-based antigen retrieval was carried out in a freshly prepared 0.01 mol/L sodium citrate buffer (pH = 6.0) solution at 750 W for 3 cycles of 10 min each. The specimens were stained with primary antibodies including CD3, CD20, CD9, CD138 and CD68 (see Table 4). Slides were counterstained with Mayer’s hematoxylin, dehydrated in alcohol, cleared in xylene, and cover slipped.

Immunohistochemical scoring: we used negative controls with brain tissue for CD3, CD20, CD138 and CD68 antibodies and healthy liver cells for CD9 marker. For positive control tonsilla was used. All positive controls were stained correctly during the procedure. None of the negative control samples showed any antigen expression. Photomicrographs were taken with Kappa image-based software (KAPPA ImageBase 2.5) using an Axiolab microscope (Zeiss, Oberkochen, Germany).

### 4.3. Statistical Data Analysis

Descriptive statistics were calculated to summarize the demographic and clinical characteristics of the study cohort. Continuous variables were expressed as the mean ± standard deviation (SD), while categorical variables were presented as absolute frequencies and percentages. Differences in baseline characteristics (age, gender, localization, and grade of dysplasia) between clinical types (homogeneous vs. non-homogeneous) were assessed using the independent-samples *t*-test for continuous data and Pearson’s chi-squared test for categorical data. A General Linear Model (GLM) coupled with non-parametric bootstrapping was conducted to ensure robust inference. This approach avoids the strict normality assumptions required by classical parametric tests and provides more reliable confidence intervals for smaller datasets. The immunohistochemical infiltration scores ranged from 1 to 4 and were derived from prespecified semi-quantitative intervals: score 1, 0–5%; score 2, 6–10%; score 3, 11–15%; score 4, 16–20%. Because these categories were defined using approximately equal percentage intervals, the scores were treated as approximately continuous for the purpose of the GLM analysis. For each marker (CD3, CD20, CD138, and CD68), a separate GLM was constructed. The model specification included fixed factors: dysplasia status (yes vs. no) and clinical form (homogeneous vs. non-homogeneous); the interaction term dysplasia × clinical type of OL bootstrap resampling (1000 iterations) was used to derive confidence intervals for parameter estimates. For pairwise post hoc comparisons, Bonferroni-adjusted *p*-values were used. Model goodness-of-fit was assessed using overall model significance and effect size estimates (partial η^2^). Upon the detection of significant interaction effects, a simple effects analysis was conducted. This involved decomposing the interaction to evaluate the effects of dysplasia specifically within the homogeneous and non-homogeneous oral leukoplakia separately. The influence of anatomical localization (buccal mucosa, lateral border of tongue, floor of mouth) on marker expression was assessed using the Kruskal–Wallis H test. Differences in the mean count of CD9+ connective tissue cells and the number of CD9+ epithelial layers between dysplastic and non-dysplastic tissues were assessed using the independent samples *t*-test. Equality of variances was verified using Levene’s test; in cases where the assumption of homogeneity of variances was violated, Welch’s *t*-test was applied. Furthermore, the linear relationship between epithelial and stromal CD9 expressions within each diagnostic group was quantified using Pearson’s correlation to determine whether the coordinated regulation of this marker was altered during dysplastic transformation. To validate the reliability of the findings, a post hoc power analysis was performed based on the observed effect sizes derived from the GLM results. The statistical data analysis was conducted with Jamovi (v.2.7). The results were considered statistically significant at a *p*-value < 0.05.

## 5. Conclusions

The present immunohistochemical study found clear differences in lamina propria cell density between homogeneous and non-homogeneous oral leukoplakia. The infiltrate consisting of plasma cells (CD138+) and macrophages (CD68+) was significantly higher in both clinical types of OL only when dysplasia was demonstrated. CD3 and C20 antigen-labeled lymphocyte density showed no statistical significance; future research should examine T- and B-lymphocyte subtypes in OL more closely. Assessing the significance of the CD138 and CD9 antigens in predicting the course of oral leukoplakia requires a parallel analysis of the expression of both markers in epithelial and connective tissue cells beneath the basement membrane. We emphasize that, during the development of high-grade dysplasia, changes in non-homogeneous OL tetraspanin occur simultaneously in both epithelial and mesenchymal cells of the lamina propria. In contrast, as leukoplakia progresses toward malignancy, CD138 antigen expression decreases in the epithelium but increases in the cells of the lamina propria.

From a clinical point of view, our study helps directly in the evaluation of OL biopsies. Dense infiltration of T lymphocytes, CD68+ macrophages and CD138+ plasma cells in a non-homogeneous OL is also an indicator for total surgical excision of leukoplakia, without waiting for the further progression of its high-grade dysplasia. If future immunohistochemical studies and literature reviews verify that CD68 and CD138 markers are present at higher levels in non-homogeneous OL lamina propria cells, this knowledge may help inform local immunotherapy as an option when leukoplakia cannot be surgically excised.

## Figures and Tables

**Table 1:** Demographic and clinical characteristics of the study group.

Characteristic	Total (N = 50)	Homogeneous (N = 18)	Non-Homogeneous (N = 32)	*p*-Value
Age, years (mean ± SD)	57.0 (±14.1)	54.8 (±13.7)	58.2 (±14.5)	0.41
Gender, n (%)				
Male	29 (58.0%)	9 (50.0%)	20 (62.5%)	0.575
Female	21 (42.0%)	9 (50.0%)	12 (37.5%)	
Localization, n (%)				
Buccal mucosa	18 (36.0%)	8 (44.4%)	10 (31.2%)	0.194
Lateral border of tongue	17 (34.0%)	4 (22.2%)	13 (40.6%)	
Floor of mouth	11 (22.0%)	3 (16.7%)	8 (25.0%)	
Other (lip, alveolar ridge)	4 (8.0%)	3 (16.7%)	1 (3.1%)	
Dysplasia status, n (%)				
Presence	33 (66.0%)	7 (38.9%)	26 (81.2%)	0.006

**Table 2:** General Linear Model results evaluating the effects of dysplasia and clinical type of oral leukoplakia on immune marker expression, adjusted for age and gender.

Marker	Parameters	df	F	*p*	Partial *η*^2^
CD3	Model	5	12.57	<0.001	0.588
	Clinical type	1	14.39	<0.001	0.246
	Dysplasia	1	9.14	0.004	0.172
	Clinical type x dysplasia	1	8.95	0.005	0.169
	Age	1	0.00	0.974	0.000
	Gender	1	0.00	0.966	0.000
CD20	Model	5	14.27	<0.001	0.618
	Clinical type	1	8.37	0.006	0.160
	Dysplasia	1	19.00	<0.001	0.302
	Clinical type x dysplasia	1	8.87	0.005	0.168
	Age	1	0.11	0.747	0.002
	Gender	1	0.06	0.811	0.001
CD138	Model	5	33.95	<0.001	0.794
	Clinical type	1	10.54	0.002	0.193
	Dysplasia	1	72.94	<0.001	0.624
	Clinical type x dysplasia	1	9.96	0.003	0.185
	Age	1	0.70	0.407	0.016
	Gender	1	0.09	0.768	0.002
CD68	Model	5	30.47	<0.001	0.776
	Clinical type	1	12.47	<0.001	0.221
	Dysplasia	1	57.08	<0.001	0.565
	Clinical type x dysplasia	1	12.93	<0.001	0.227
	Age	1	0.00	0.964	0.000
	Gender	1	0.16	0.687	0.004

**Table 3:** Simple effects analysis decomposing the significant clinical type of oral leukoplakia x dysplasia interaction for each marker. Note: All General Linear Models used 1000 bootstrap resamples for estimating parameters. CI = confidence interval. Estimates represent the mean difference in expression levels (dysplasia vs. no dysplasia) within the specified clinical type of oral leukoplakia.

Marker	Oral Leukoplakia: Clinical Types	Effect of Dysplasia (Estimate)	95% CI	*p*
CD3	Homogeneous	0.043	−0.363–0.419	0.894
	Non-homogeneous	1.312	0.922–1.667	<0.001
CD20	Homogeneous	0.419	0.006–0.951	0.284
	Non-homogeneous	1.952	1.487–2.383	<0.001
CD138	Homogeneous	0.999	0.800–1.182	<0.001
	Non-homogeneous	2.075	1.758–2.385	<0.001
CD68	Homogeneous	0.885	0.509–1.178	0.005
	Non-homogeneous	2.336	1.953–2.656	<0.001

**Table 4:** Antibody clones, manufacturers, and dilutions.

Antibody	Clone	Manufacturer	Dilution
CD3	F7.2.38	DAKO (Glostrup, Denmark)	Ready-to-use
CD20	L26	DAKO (Glostrup, Denmark)	Ready-to-use
CD138	M15	DAKO (Glostrup, Denmark)	Ready-to-use
CD68	KP1	DAKO (Glostrup, Denmark)	Ready-to-use
CD9	No. 4H7B9	Proteintech (Rosemont, IL, USA)	1:1000

## Data Availability

The original contributions presented in this study are included in the article/Supplementary Materials. Further inquiries can be directed to the corresponding author.

## Supplementary Materials

The following supporting information can be downloaded at: https://www.mdpi.com/article/10.3390/ijms27146111/s1.
