# Supplementary material for: Immunological Microenvironment Diversity in Homogeneous and Non-Homogeneous Oral Leukoplakia
Source: Int J Mol Sci. 2026 Jul 8;27(14):6111. doi: 10.3390/ijms27146111 (PMC13410727; doi:10.3390/ijms27146111)
Supplement: Supplementary file 1 [file ijms-27-06111-s001.zip › Table S2.pdf]

Table S2.

Immunological microenvironment diversity in oral leukoplakia during its precancerous stages

Ingrīda Čēma, Regīna Kleina, Madara Dzudzilo, Kristina Lasiené, Anita Dabužinskiene, Julianna Muceniece, Maksims Zolovs, Tālivaldis Freivalds

Table shows patient clinical data—including the type of OL (homogenous and non-homogenous, presence or lack of dysplasia, age, sex and location of 50 oral leukoplakia. Immunohistochemical results about immune cells beneath OL are demonstrated by CD3, CD 20, CD 68 and CD 138 antigens. The density of lamina propria cell infiltration had four scores: score 1- (0-5), score 2- (6-10), score 3- (11-15) and score 4- (16-20).
