# Supplementary material for: Immunological Microenvironment Diversity in Homogeneous and Non-Homogeneous Oral Leukoplakia
Source: Int J Mol Sci. 2026 Jul 8;27(14):6111. doi: 10.3390/ijms27146111 (PMC13410727; doi:10.3390/ijms27146111)
Supplement: Supplementary file 1 [file ijms-27-06111-s001.zip › Table S1.pdf]

Table S1.

Immunological microenvironment diversity in oral leukoplakia during its precancerous stages

Ingrīda Čēma, Regīna Kleina, Madara Dzudzilo, Kristina Lasiené, Anita Dabužinskiene, Julianna Muceniece, Maksims Zolovs, Tālivaldis Freivalds

The table shows the personal data of 50 patients with the first letters of the surname and first name. Oral leukoplakias are divided into two groups: with and without dysplasia in them. Accordingly, the number of epithelial layers labeled with CD9 antigen and lamina propria mesenchymal cells under the basement membrane at 400x magnification has been assessed in each leukoplakia. The results are shown in whole numbers
